# An ethnobotanical study on the medicinal herb practices of the gelao ethnic minority in North Guizhou, China: an exploration of traditional knowledge

**DOI:** 10.3389/fphar.2023.1217599

**Published:** 2023-08-31

**Authors:** Fusong Liu, Jie Peng, Yi Feng, Yuhan Ma, Yan Ren, Pei Sun, Yongxia Zhao, Sha Liu, Faming Wu, Jian Xie

**Affiliations:** ^1^ School of Pharmacy, Zunyi Medical University, Zunyi, China; ^2^ School of Pharmacy, Southwest Minzu University, Chengdu, China; ^3^ Industrial Crop Research Institute, Sichuan Academy of Agriculture Sciences, Chengdu, China; ^4^ Guizhou Medical and Health Industry Research Institute, Zunyi Medical University, Zunyi, China

**Keywords:** gelao ethnic minority, traditional medicinal herbs, ethnobotany, wild plants, NCSI

## Abstract

**Introduction:** The Gelao ethnic minority of northern Guizhou, China have long possessed extensive traditional knowledge of medicinal herbs. This ethnobotanical study aimed to document and evaluate wild plants used medicinally by the Gelao people, providing insights into their traditional medicine and knowledge systems.

**Methods:** Field research was conducted in Gelao communities of Daozhen, Wuchuan and Zheng’an counties using interviews, surveys and participatory rural appraisal.

**Results:** Quantitative ethnobotanical indices were utilized to assess the cultural significance of 187 herbs identified. The herbs belonged to 84 families, primarily Compositae, and were mostly roots, rhizomes and whole plants. They were used to treat digestive, respiratory and inflammatory disorders, gynecological diseases, bites and other conditions, mainly through decoctions. 25 highly significant herbs (national plant cultural significance index > 1000) were known to protect health. Some function as food and are considered safe. However, the study revealed issues including a declining number of knowledgeable elders and inadequate hygiene controls.

**Conclusion:** Our findings demonstrate the Gelao’s extensive medicinal plant knowledge and highlight the need for further ethnobotanical research to document and preserve this culturally important tradition. The identified herbs also represent an alternative medicinal resource with potential modern applications pending further investigation of their pharmacology and sustainable use. Overall, this study provides valuable insights into Gelao ethnobotanical knowledge and the potential of indigenous medicine for modern healthcare.

## Introduction

Throughout history, the human species has harnessed the bounty of natural resources to combat diseases, leading to the evolution of diverse traditional medicinal cultures, each with distinct regional characteristics (Jia et al., 2015). However, the swift advancement of modern medicine has precipitated the decline, and in some cases, the extinction of these traditional medicinal cultures, thereby dealing a significant blow to our collective human heritage (Li et al., 2015; Yang et al., 2021). China is a country with one of the best-preserved traditional medicinal cultures, with traditional Chinese medicine being fully inherited and developed to play an unimaginable role in disease prevention, treatment, and daily healthcare (Wang, 2013). In addition, other traditional medicinal cultures, such as Tibetan medicine, Mongolian medicine, Uyghur medicine, Dai medicine, and Miao medicine, have also been well-preserved and developed (Sun et al., 2020). Almost every ethnic minority in China boasts a unique medicinal culture, contributing significantly to their ethnic inheritance (Kang et al., 2016; Li et al., 2020; Zi and Zeng, 2023).

Guizhou, a province populated by a tapestry of ethnic groups, is home to the Gelao ethnic minority, an indigenous community with a rich and unique cultural heritage. Over 90% of the global Gelao population resides in the Zunyi region of northern Guizhou, notably in Wuchuan County and Daozhen County—Gelao autonomous counties where approximately half of the inhabitants are of Gelao descent. The Gelao people, steeped in history, have crafted a vibrant and unique culture, with traditional Gelao medicine serving as a key pillar. This ancient and enigmatic community has a wealth of knowledge and practices concerning medicinal plants, utilizing at least 61 species known for their edibility and medicinal properties. The book “Gelao Medicine” records 200 types of therapeutic agents commonly harnessed by the Gelao community, including 174 plant-based, 23 animal-based, and 3 mineral-based remedies. The roots of Gelao traditional medicine stretch back to at least 2200 years ago, the Han dynasty era, as evidenced by 63 of their commonly used remedies being recorded in the ancient Chinese pharmacopoeia, “Shennong Bencaojing” (The Classic of Herbal Medicine). While it is impossible to ascertain who first utilized these substances medicinally, Gelao medicine exhibits distinct characteristics when compared to traditional Chinese medicine and other ethnic traditional medicines, especially in the application of the same remedy for different ailments, and the combination and compatibility of remedies. The Gelao traditional medicine philosophy is grounded in the concept of yin-yang balance, which posits the existence of a life force called ‘qi’ flowing through invisible meridians in the body. Disruptions in the flow and distribution of qi are believed to result in disease, and treatments aim to restore the balance and harmony of qi through various methods. The therapeutic repertoire of Gelao traditional medicine is diverse, encompassing external therapies like acupuncture, moxibustion, cupping, and massage, and internal therapies involving decoctions, wines, powders, and pills made from various plant or animal materials.

However, the Gelao people have not formed a systematic script, and their traditional culture has primarily been preserved through oral transmission, inscriptions, and documents, which continue to be passed down to this day. Unfortunately, the traditional Gelao medicine culture is rapidly disappearing due to the influence of modern medicine, traditional Chinese medicine, as well as factors such as ethnic Hanization, relocation, and poverty alleviation. In terms of ethnic ethnobotanical research in Guizhou, most studies have focused on investigation and classification of edible, tea-like, and dyeing ethnic plants, such as He’s research on the edible plants of the Dong ethnic group (He et al., 2019), Hong’s research on the Shui ethnic group (Hong et al., 2015a), and Hu’s research on the Baiku Yao ethnic group (Hu et al., 2022). These studies have found that different ethnic groups have rich and diverse plants with unique cultural characteristics, providing valuable information for the research and development of ethnic plants in Guizhou. Our previous study also found that the Gelao people have rich traditional knowledge of wild edible plants (Xie et al., 2022). There is a lack of research focusing on traditional medicinal knowledge of ethnic herbs in Guizhou, particularly concerning traditional Gelao medicinal herbs. This study aims to address this gap.

Therefore, this study employed ethnobotanical research methods (Wang, 2017) to investigate the traditional medicinal herbs of the Gelao ethnic minority. Our objective is to understand their traditional knowledge of herb usage in specific regions and groups, the relationship between their villages and natural environments, and the use of traditional medicinal herbs in local community health practices. This study represents the first comprehensive ethnobotanical investigation and documentation of the traditional medicinal plants used by the Gelao ethnic minority in northern Guizhou, China, filling a significant research gap in this field. We utilized various quantitative and qualitative methods to conduct in-depth analysis of the diversity, selection, conservation value, and other aspects of the Gelao traditional medicinal plants, revealing the characteristics and patterns of the Gelao traditional medicinal plant knowledge. Furthermore, we combined the historical, cultural, and ecological background of the Gelao ethnic group, and explored the formation, development, and changes of the Gelao traditional medicinal plant knowledge, providing a new perspective for understanding and protecting the Gelao ethnobotany and ethnic culture. This study will also offer appropriate suggestions for the sustainable use of plant resources for traditional Gelao medicine.

## Materials and methods

### Study area

**FIGURE 1:**
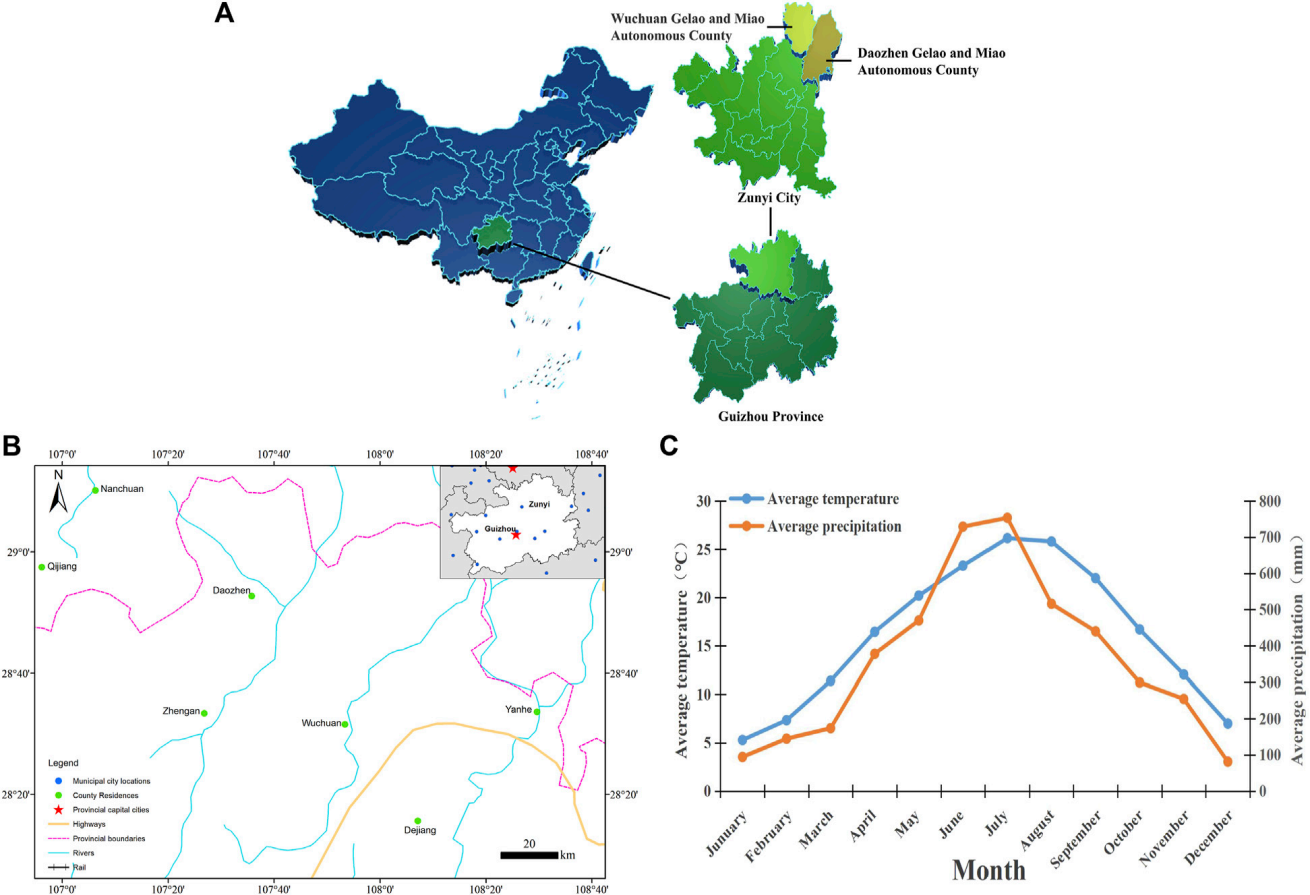
Survey area. **(A)** Daozhen County, Wuchuan County and Zheng’an County belong to a minority autonomous county in the northeastern mountainous area of Guizhou Province. **(B)** The traffic situation in the study area. **(C)** The annual average temperature and precipitation.

The present study was conducted in the northern part of Guizhou province, specifically in Daozhen, Wuchuan, and Zheng’an counties. These counties are situated between 28°9′-29°13′N and 107°4′-108°13′E, encompassing the Dalou Mountains, southeastern foothills, and the branch and sub-branches of the Dalou Mountain Range, as well as the upper reaches of the Furong River. The region experiences a subtropical humid monsoon climate and a mid-subtropical humid monsoon climate, with an average annual temperature of 8°C–16.14°C and an annual precipitation of 800–1400 mm (Figure 1) (Yang et al., 2002). The local economy primarily relies on agriculture, with crops such as rice, corn, vegetables, and tea oil being the main agricultural products. Additionally, tourism plays a significant role in the region, attracting a large number of visitors.

The study area is a diverse settlement comprising multiple ethnic groups, including the Gelao, Miao, and Han ethnic groups. Daozhen and Wuchuan serve as the birthplace and main settlement area of the Gelao people, with a population of 310,000 (Xiao et al., 2022). The Gelao people have created various spiritual and cultural heritages throughout their historical practice and passed them down from generation to generation, forming the unique intangible cultural heritage of the Gelao people. The “Nuo” culture represents a typical aspect of their culture, originating from the totem worship of primitive society and belonging to the category of shamanism culture. The ancestors of the Gelao people held primitive religious beliefs that “all things have spirits” and developed shamanic activities that revolved around daily life. This gave rise to figures such as the “Duan Gong” and “witch,” who served as intermediaries between humans and gods, driving away evil forces and protecting human beings. Additionally, the Gelao people celebrate various traditional festivals that showcase their unique characteristics. These include the Mountain Worship Festival, the New Rice Festival, and the Cattle Worship Festival. The Mountain Worship Festival takes place on the third day of the third lunar month each year, during which people worship mountains or trees. The New Rice Festival, held in July or August depending on the maturity of early rice, commemorates the contributions of ancestors in opening up new farmland and celebrates the upcoming harvest. The traditional Cattle Worship Festival, celebrated on the first day of October in the lunar calendar, originated from the Gelao people’s domestication of wild buffaloes, which eventually became domestic cattle, and their subsequent worship of these animals (Shang, 2017). These traditional cultures and festivals highlight the Gelao people’s traditional way of life and unique cultural characteristics.

### Ethnobotanical information collection

During the survey, we employed various methods to collect ethnobotanical information. Key informant interviews, semi-structured interviews, and participatory observation were utilized (Cheng et al., 2020), with interviews based on the “5W+1H” framework, which includes questions about who, what, where, when, why, and how (Pei and Long, 1998).

Key informant interview is a qualitative research method used to collect traditional knowledge from local residents who possess expertise and experience, such as barefoot doctors and professional herbalists. In this study, we conducted in-depth interviews with representative individuals from three generations of the Gelao ethnic group, including elders with herbal knowledge, middle-aged people, and young individuals with some experience in herbal use. We collected information on medicinal plants and recorded, organized, and analyzed the traditional knowledge of herbal medicine used by the Gelao people. This included information such as local names, medicinal parts, processing methods, efficacy, safety, and other basic details.

**FIGURE 2:**
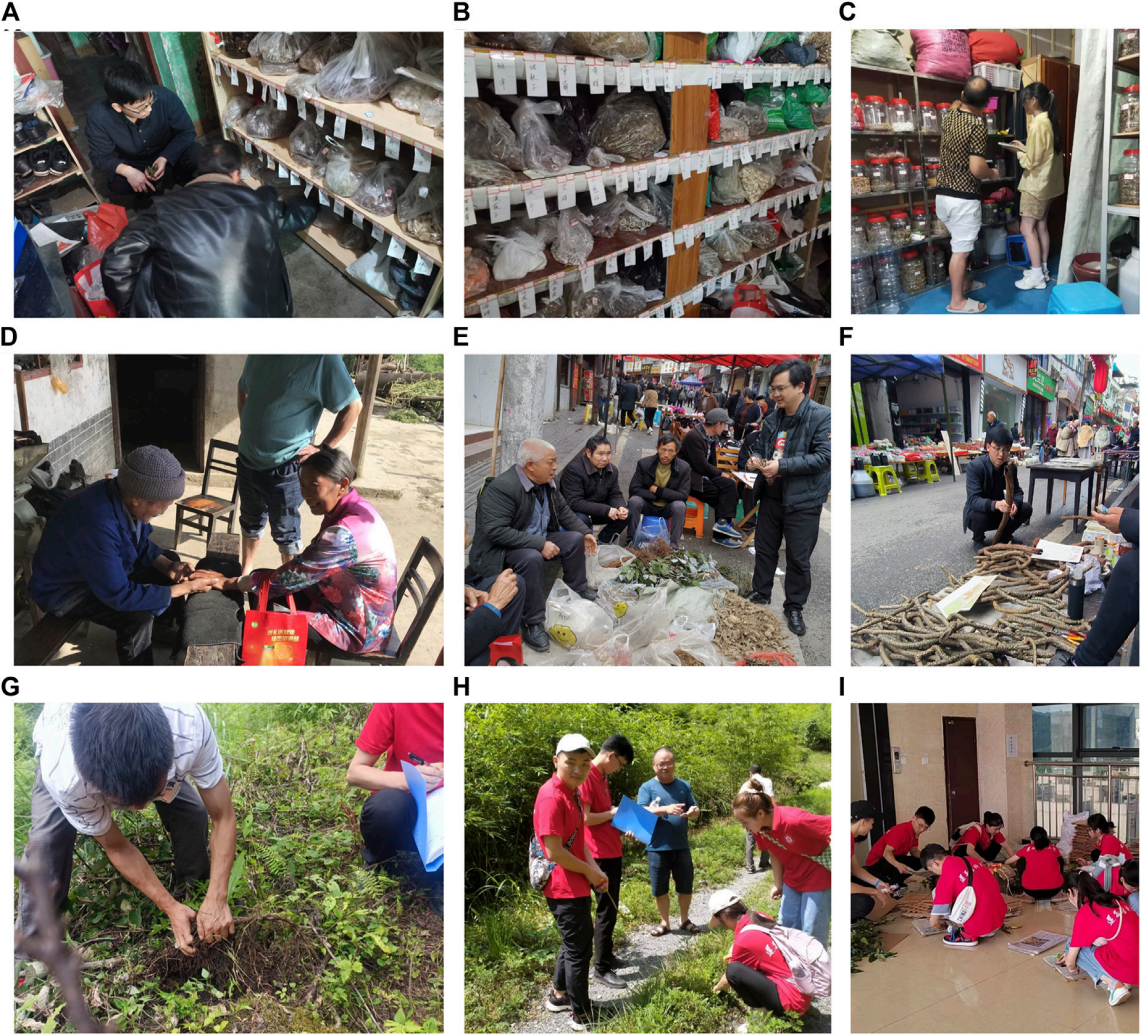
Ethnobotanical investigation of Gelao Nationality. **(A–C)** Surveys at the local herbal shop. **(D)** A doctor of Gelao nationality is diagnosing the patient. **(E, F)** Surveys in local temporary markets. **(G, H)** Collect medicinal specimens under the guidance of local people. **(I)** Making medicinal specimens.

Semi-structured interviews, on the other hand, involve a predetermined list of questions to gather qualitative data. This approach can provide more specific and detailed information (Supplementary Table S1). We conducted surveys on herbal medicine in herbal shops and temporary markets in the local area. Temporary markets are organized by local residents every 5 days, with different towns staggering their market days. For example, town A holds markets on the first, sixth, 11th, 16th, 21st, and 26th days of each month, while town B holds markets on the second, seventh, 12th, 17th, 22nd, and 27th days of each month. These temporary markets effectively bring together local herbal doctors and Gelao residents who purchase herbs. To gather a large amount of information in a limited time, we distributed survey documents (Figures 2A–F).

Participatory Rural Appraisal (PRA) is a widely used method in ethnobotanical research, in which researchers and local experts with abundant knowledge of local plants, such as doctors or farmers, investigate the overall situation of medicinal plants and related issues to explore and solve problems. We learned how Gelao people identify and distinguish medicinal plants from local herbal doctors or farmers, and we acquired knowledge about the methods of collecting, processing, and preserving herbs. With the guidance of these experts, we collected local herbs, made herbal specimens, and recorded information such as the growth environment, collection location, and time of the herbs (Figures 2G,H). Combining literature and professional knowledge, we classified and identified the herbs, determined their plant sources, recorded their medicinal value, usage, and dosage, and brought plant specimens back to the laboratory for further analysis (Figure 2I).

### Ethnobotanical quantitative evaluation method

The national plant cultural significance index (NCSI) was used to quantitatively evaluate the wild medicinal plants in the surveyed area. The evaluation was based on the following formula:

$$NCSI=FQI\times AI\times FUI\times PUI\times MFI\times CEI\times DSI\times 10^{-2}$$

where FQI represents the frequency of quotation index, AI represents the availability index, FUI represents the frequency of utilization index, PUI represents the parts used index, MFI represents the multifunctional use index, CEI represents the curative effect index, and DSI represents the drug safety index (Pieroni, 2000).

The values for each index were established and assigned levels based on the guidelines outlined in “Research Methods in Ethnic Botany” (Wang, 2017). The frequency of quotation index (FQI) refers to the number of individuals who mention a particular plant among all information sources. The availability index (AI) is divided into very common (4.0), common (3.0), ordinary (2.0), and uncommon (1.0). The frequency of utilization index (FUI) is categorized as more than 10 times per year (5.0), 6–10 times per year (4.0), 2–5 times per year (3.0), at least once per year (2.0), once every 2–3 years (1.0), and not used in the past 5 years (0.5). The parts used index (PUI) is classified as whole plant (5.0), aboveground or underground parts (4.0), stems, leaves, flowers, fruits, seeds (3.0), bark, kernels (2.0), special parts, processed products (1.0). The multifunctional use index (MFI) has a base value of 0 and increases by 1 for each additional use, with a score of 1 for only one use and 5 for five uses. The curative effect index (CEI) is divided into excellent (5.0), very good (4.0), good (3.0), fair (2.0), and poor (1.0). The drug safety index (DSI) is categorized as very high (medicinal and edible: 5.0), high (safe and non-toxic: 4.0), moderately high (has some side effects: 3.0), moderate (slightly toxic: 2.0), and low (highly toxic: 1.0).

### Specimen identification

During the course of this survey, we identified the plant species by referring to various botanical literature, including the electronic version of the “Flora of China” (http://www.iplant.cn/frps) (Qian, 1963), the “Illustrated Handbook of Flowers in Hengduan Mountains” (Niu and Sun, 2021), and the “Field Identification Handbook of Common Plants in China: Hengshan Volume” (Ma and He, 2016). The identification process involved observing morphological characteristics, leaves, flowers, fruits, and other parts of the plants, and comparing them with relevant botanical literature to determine their species classification. We then prepared herbarium specimens, organized and analyzed all collected information according to the research objectives, and created tables and charts. The relevant specimens collected during the survey have been deposited in the Chinese Medicinal Herbarium at the School of Pharmacy, Zunyi Medical University.

## Results

### Basic information of informants

**FIGURE 3:**
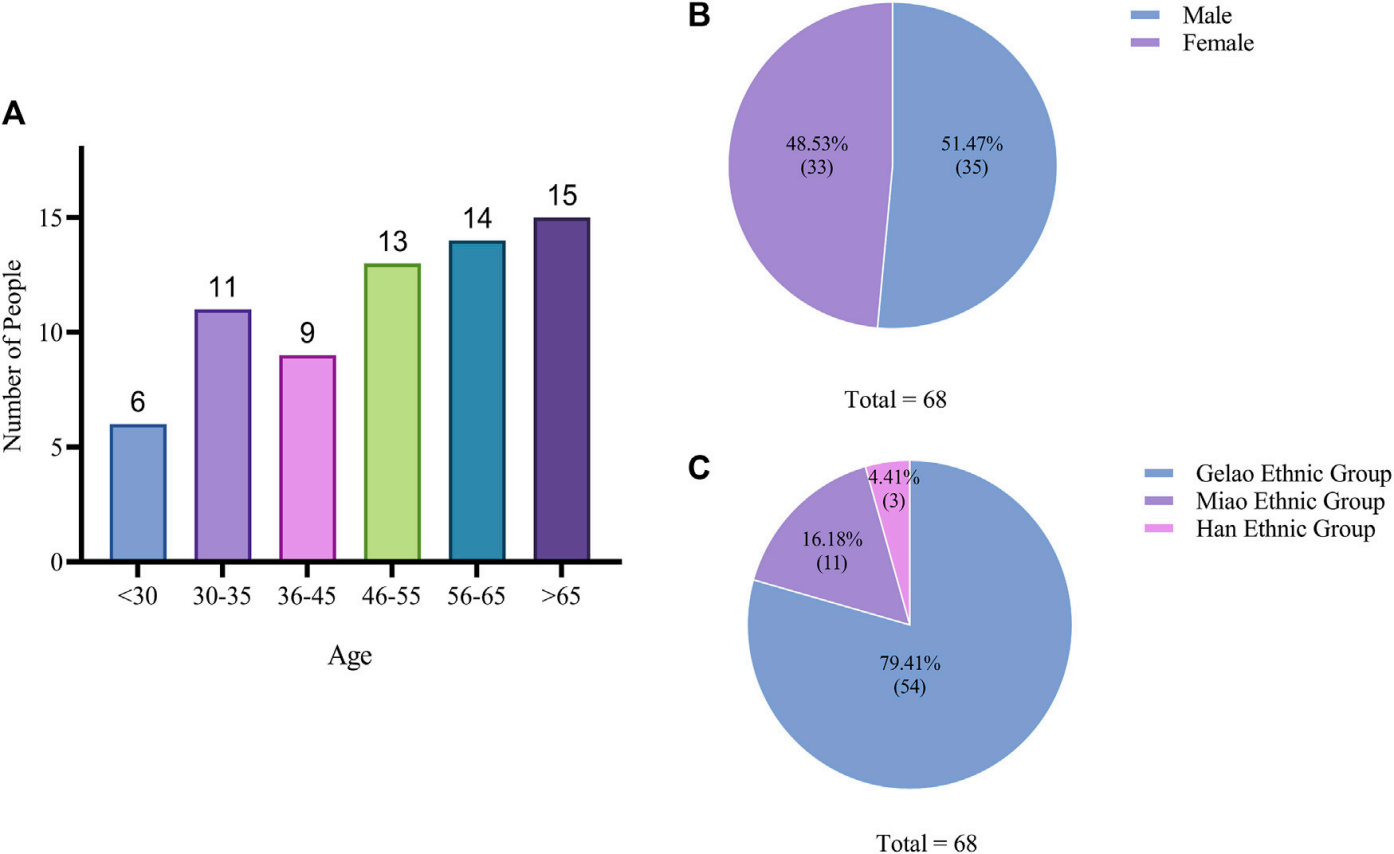
Demographic profile of informants.

The basic information of the 68 informants was statistically analyzed. The results showed that the ages of all informants ranged from 24 to 89 years, with 6 informants under 30 years old, 11 between 30 to 35 years old, 9 between 36 to 45 years old, 13 between 46 to 55 years old, 14 between 56 to 65 years old, and 15 over 65 years old. Of the total informants, 35 were male and 33 were female, resulting in an almost equal gender ratio. Among them, 54 were from the Gelao ethnic group, accounting for 79.41% of the total, while 11 were from the Miao ethnic group and 3 were from the Han ethnic group. The survey results indicated that informants over the age of 50 were more likely to provide effective information, especially as young people below the age of 35 were unable to provide much useful information (Figure 3).

### History and cultural background of herbal use among the gelao ethnic group

The Gelao ethnic group, residing primarily in the mountainous areas of northern Guizhou in southern China, possesses a rich cultural heritage and traditional medicinal knowledge, with herbal medicine playing a significant role in their daily lives. Herbal medicine serves as a traditional healing method and is deeply rooted in their cultural practices. Existing literature indicates that the Gelao people have a long history of herbal medicine use, with their knowledge, utilization, and transmission of herbal medicine intertwined within their social fabric (A, 2006). Within the Gelao’s traditional medical system, herbal medicine holds paramount importance, believed to treat physical illnesses, regulate health, and even ward off malevolent spirits.

Survey results show that the Gelao people’s knowledge of traditional medicine is passed down through oral transmission of experience and practice, resulting in unique regional and familial inheritance characteristics in the use and dissemination of herbal medicine (Wei et al., 2002). The Gelao employ various forms of herbal medicine preparations, including decoctions, tinctures, powders, external applications, fumigants, and more. Specific rules govern the combination and compatibility of different herbal medicines for different ailments (Zhao, 2003). For example, *Taraxacum mongolicum* Hand.-Mazz., *Paris polyphylla* Sm., and *Rostellularia mollissima* (Nees) Nees are widely used herbal medicines among the Gelao, known for their heat-clearing, detoxifying, anti-inflammatory, and analgesic properties. Apart from their medical applications, the Gelao incorporate herbal medicine into various aspects of their daily lives. In food preparation, herbs such as *Jasminum sambac* (L.) Aiton, *Capsicum annuum* L., and *Zingiber officinale* Roscoe are used to add flavor and medicinal value to their cuisine. Adding herbal medicine to soups enhances their medicinal properties. Herbal medicine is also utilized for dye-making (*Rubia cordifolia* L.), spices (*Cinnamomum cassia* (L.) J.Presl), skincare products (*Z. officinale* Roscoe, *Litsea coreana* var. *lanuginosa* (Migo) Yen C.Yang & P.H.Huang).

**FIGURE 4:**
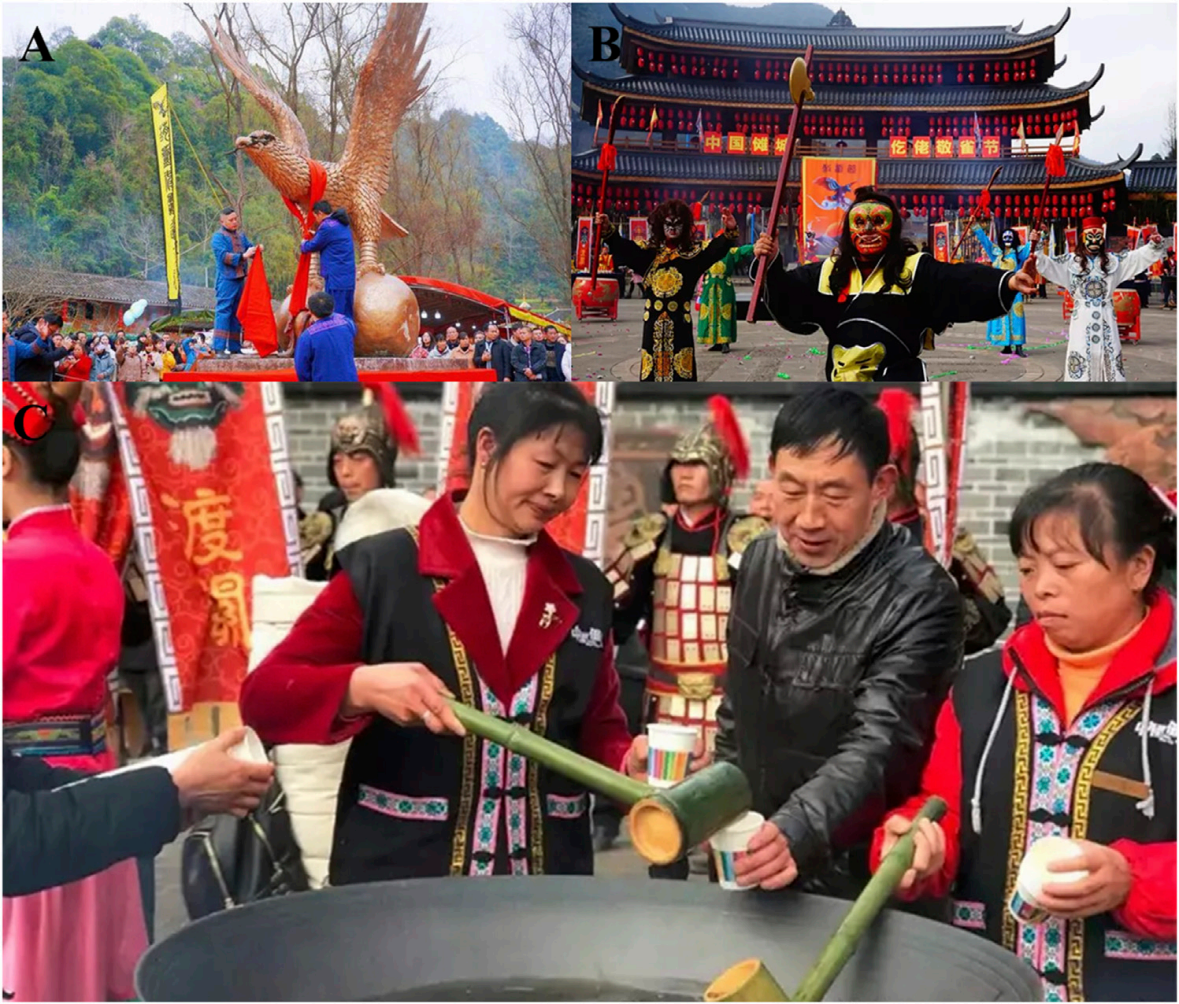
The Gelao ethnic group celebrates the “Respectful Sparrow Festival”. **(A)** A ceremony where they pay homage to their totem. **(B)** During this festival, the Gelao people perform a local exorcism dance. **(C)** People consume herbal decoction at the “Respectful Sparrow Festival”. (The pictures come from https://www.baidu.com/).

Furthermore, many of the Gelao’s festivals are closely connected to the use of herbal medicine. The application and utilization of herbal medicine among the Gelao people often intertwine with their religious beliefs, which are rooted in shamanism. According to their beliefs, various supernatural forces, including gods, ghosts, and ancestral spirits, exist in nature and can influence people’s lives and health. Therefore, during the process of using herbal medicine, the Gelao people often engage in communication and prayers with these deities, seeking blessings and protection. For example, during the annual “Respectful Sparrow Festival”, herbs are collected to create sacrificial objects for ancestor worship and to ward off evil spirits (Luo, 2018). Legend has it that a severe epidemic once broke out in a Gelao village, and the ancestors searched everywhere for a cure, but to no avail. Many people fell ill and died. At this critical juncture, a divine eagle brought a divine herb, saving the lives of the entire village. This is the origin of the “Respectful Sparrow Festival”, which has been passed down for 300 years (Figure 4). This traditional festival is the only mountain king sacrifice and blessing ceremony in the southwestern region, where Gelao people gather together to drink herbal medicine to dispel poison (Zhou and Zhang, 2006).

### Sources of traditional medicinal plants used by the gelao ethnic group

**FIGURE 5:**
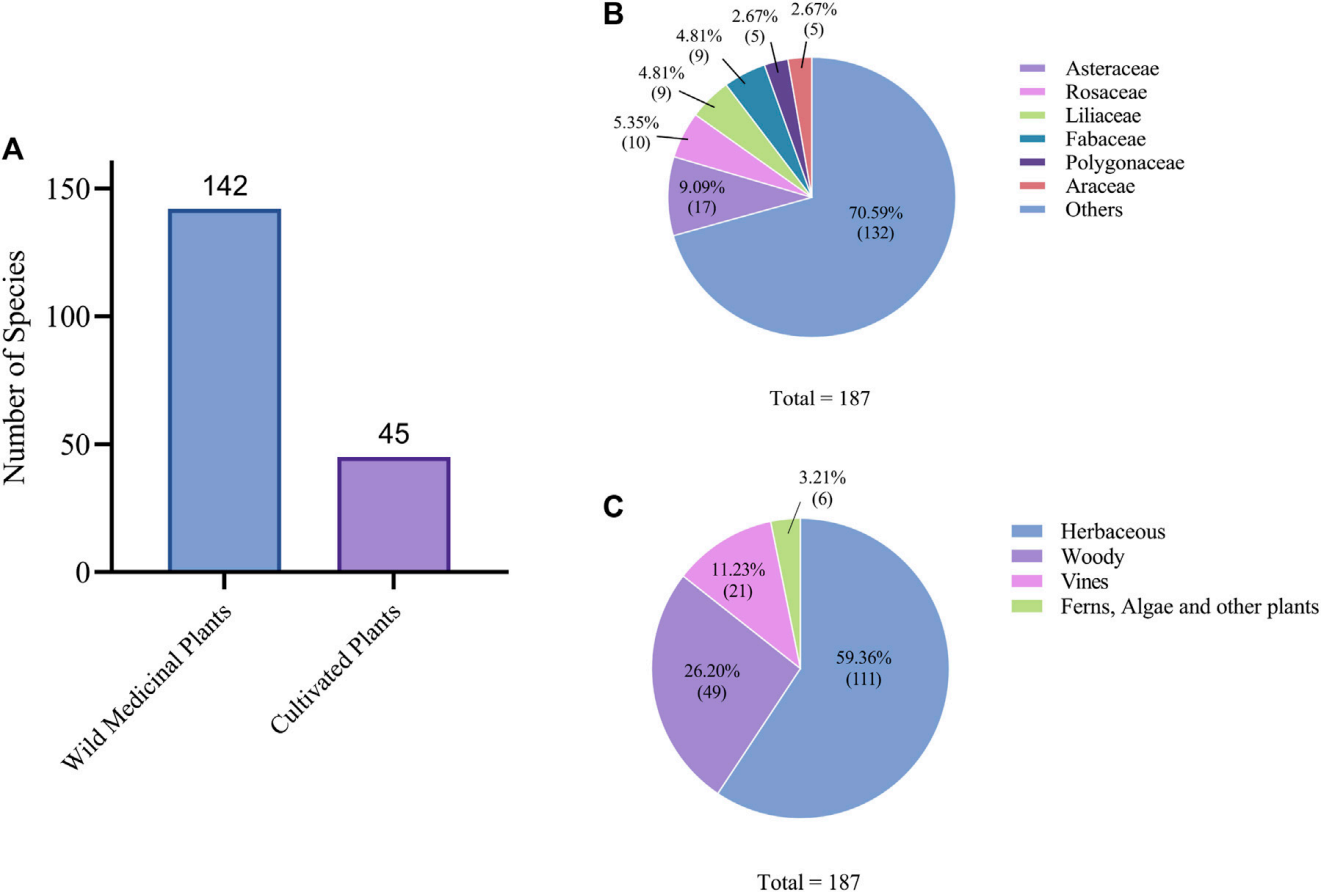
Frequency of use of the medicinal plants in the study area. **(A)** Wild medicinal plants and cultivated medicinal plants were found in the survey area. **(B)** Taxonomic diversity of medicinal plants in the study area. **(C)** Life forms of medicinal plants in the study area.

The sources of traditional Gelao medicinal plants are abundant. Our survey identified 142 species of wild medicinal plants and 45 species of cultivated plants, some of which can also be found in the wild (Figure 5A). Although cultivated plants contribute to the availability of medicinal materials, the Gelao primarily rely on collecting wild plants. The collected specimens were mainly found between altitudes of 800–2000 m in areas such as front and backyards, roadsides, nearby mountains, valleys, and farmlands.

In terms of plant taxonomy, we collected a total of 187 species of Gelao medicinal plants from 84 families. The most prominent families were Compositae, Rosaceae, Liliaceae, and Leguminosae, with 17, 10, 9, and 9 species, respectively. Additionally, the Gramineae, Umbelliferae, and Rutaceae families also had a significant presence. The Polygonaceae and Araceae families were relatively special, each with 5 species of medicinal plants (Figure 5B). In reality, there may be more species of Araceae plants, but they were difficult to distinguish during the investigation (Supplementary Table S1).

We also analyzed the botanical characteristics of the medicinal plants we surveyed. Out of the medicinal plants, 111 species were herbaceous, accounting for 59.36% of the total, 49 species were woody, accounting for 26.20% of the total, 21 species were vines, accounting for 11.23% of the total, and 6 species were ferns, algae, and other plants, accounting for 3.21% of the total (Figure 5C).

Approximately One-fourth of these medicinal plants are shade-tolerant, indicating that the selection of medicinal plant types by the Gelao people is influenced by their living environment. Some plants can yield two types of medicinal materials, such as *Reynoutria multiflora* (Thunb.) Moldenke and *Eriobotrya japonica* (Thunb.) Lindl.

### Parts used and processing methods of traditional medicinal plants used by the gelao ethnic group

**FIGURE 6:**
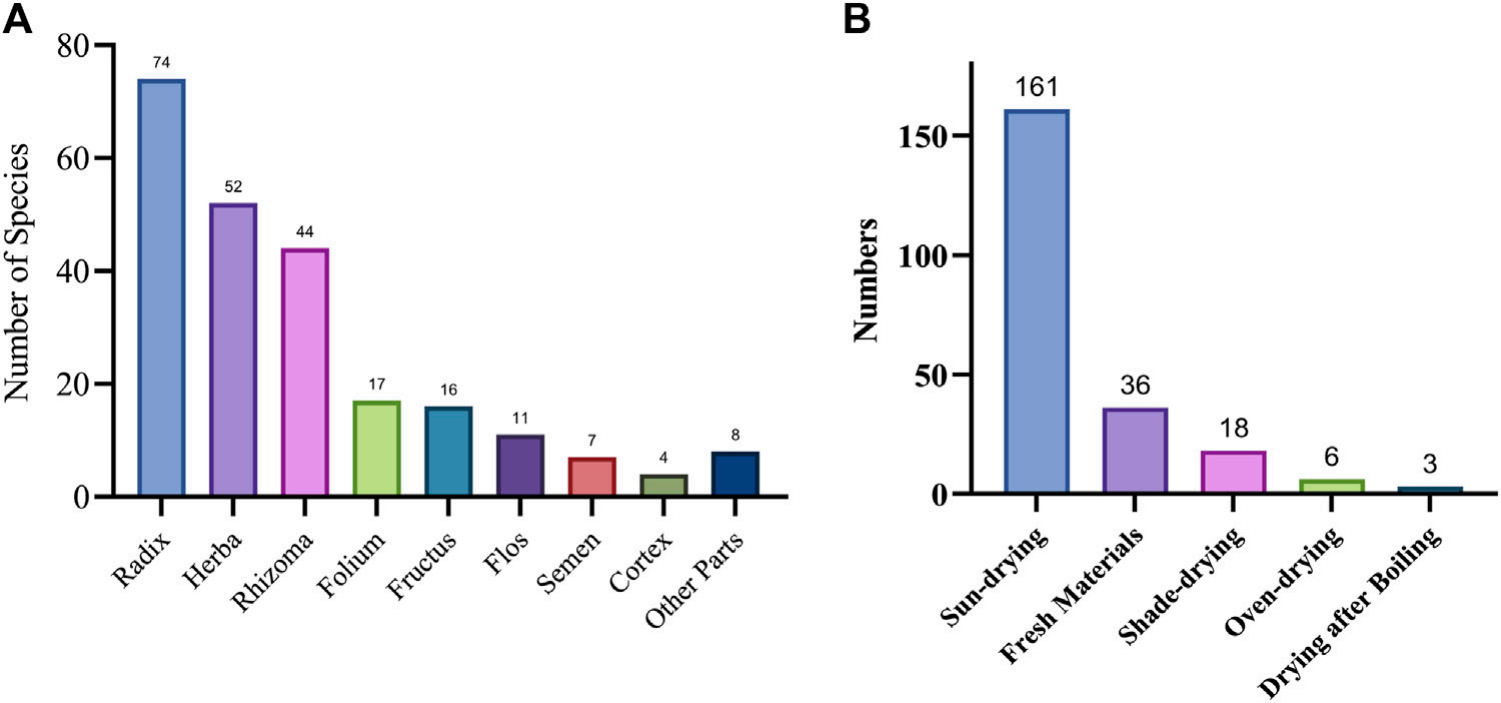
Use parts and processing methods of medicinal plants. **(A)** Plant parts used for the treatment of human ailments. **(B)** processing methods of medicinal plants.

In this study, we collected 187 traditional medicinal plants used by the Gelao ethnic group, categorized based on the plant parts utilized, including Herba (the entire plant or aboveground parts), Radix et Rhizoma (roots, rhizomes, Bulbs, and other underground parts), Fructus (fruits), Folium (leaves), and Flos (flowers). Radix-based medicinal plants were the most prevalent, with 74 species, followed by Herba-based plants (52 species). Some medicinal plants that are specified to use a particular part in the Chinese Pharmacopoeia are traditionally used by the Gelao ethnic group as Herba, such as *Lygodium japonicum* (Thunb.) Sw. There were 17 species of Folium-based medicinal plants, 16 species of Fructus-based ones, 11 species of Flos-based ones, 7 species of Semen-based ones, 4 species of Cortex-based ones, and 8 species of other parts (Figure 6A). Some of these plants have multiple parts that can be used medicinally, such as *Taxus chinensis* (Pilg.) Rehder and *Talinum paniculatum* (Jacq.) Gaertn.

Regarding processing methods, the Gelao ethnic group primarily employed simple sun-drying, which was used for 161 species. After sun-drying, some medicinal plants were cut into sections (mainly for herbal medicinal materials), while others were sliced (mainly for Radix, Caulis, and Tendrillus medicinal materials). The second most common processing method involved using fresh materials directly (36 species). The emphasis on using fresh materials is an important characteristic of the Gelao ethnic group’s traditional medicinal plants use. Shade-drying (10 species) was mainly used for certain aromatic medicinal plants, and other methods such as oven-drying and drying after boiling were also utilized (Figure 6B). The choice of processing method varied depending on the plant part used and the desired medicinal properties.

### Functions and applications of traditional medicinal plants used by the gelao ethnic group

**FIGURE 7:**
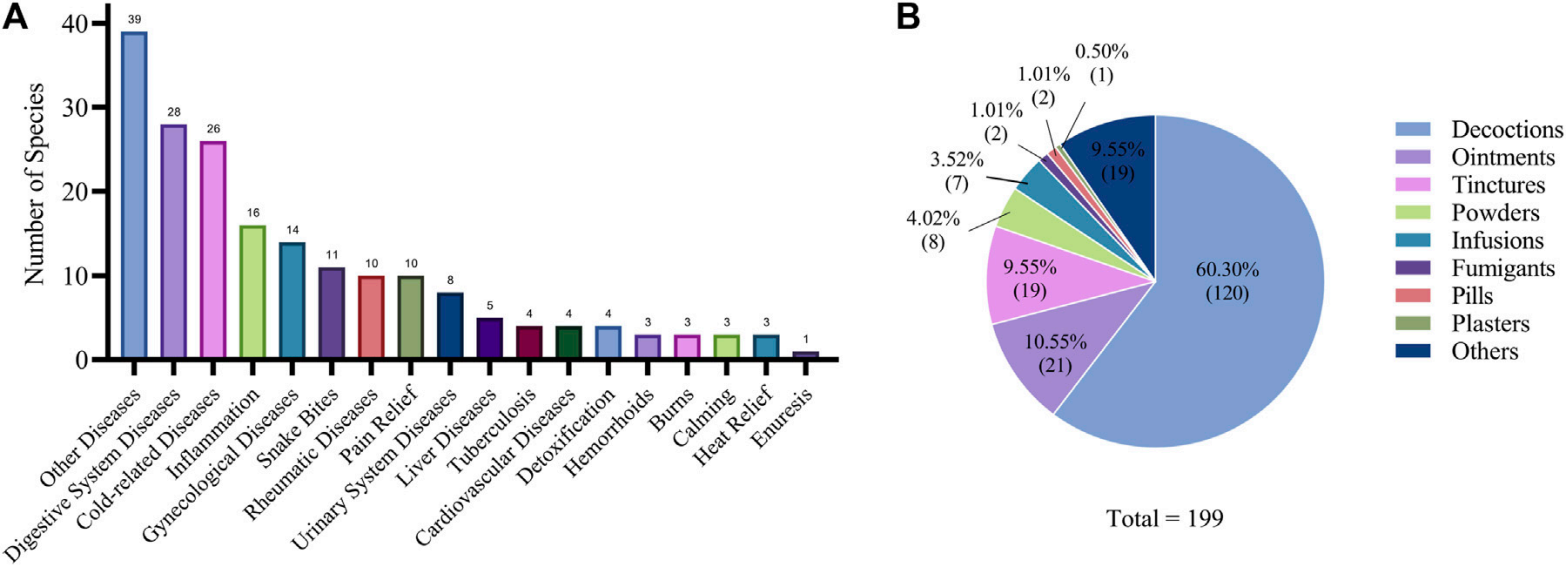
Functions and applications of traditional medicinal plants used by the gelao ethnic group. **(A)** diseases treated by Gelao medicinal plants. **(B)** The methods of using traditional medicines of the Gelao ethnic group.

The traditional medicinal plants used by the Gelao ethnic group serve various functions and applications in treating a range of diseases. Among the 187 recorded plant species, the most common applications were for treating digestive system diseases (28 species) and cold-related diseases (26 species). Additionally, there were 16 species for treating inflammation, 14 species for gynecological diseases, 11 species for snake bites, 10 species for rheumatic diseases, 8 species for urinary system diseases, 5 species for liver diseases, 4 species for tuberculosis, 4 species for cardiovascular diseases, 3 species for hemorrhoids, 3 species for burns, and 1 species for enuresis. Furthermore, there were 12 species for nourishing, 10 species for pain relief, 4 species for detoxification, 3 species for calming, and 3 species for heat relief. There were also 39 species that could not be classified into specific categories (Figure 7A).

During the investigation, it was observed that Gelao medicinal practitioners primarily prescribed treatments for digestive system diseases, particularly intestinal diseases, and rheumatic diseases, which accounted for approximately half of all prescriptions. However, the use of traditional medicine has declined. Although we found many medicinal plants that were effective for treating snake bites, they are not commonly used today. The reasons for this are twofold. Firstly, after the relocation and poverty alleviation efforts in Guizhou Province, most Gelao people have moved away from their traditional living environment, and such cases have become less frequent. Secondly, in cases of emergency and serious illnesses, people no longer seek treatment from traditional medicinal practitioners, but instead prioritize seeking advanced medical treatment.

Gelao medicinal practitioners primarily focus on treating chronic diseases, but they still play an irreplaceable role in the region during special periods. For instance, certain Gelao traditional medicinal plants were widely applied in combating COVID-19, especially for treating cough, sore throat, and diarrhea. Plants like *Stemona japonica* (Bl.) Miq, *Reineckea carnea* (Andrews) Kunth, and *Ainsliaea glabra* Hemsl. were used for cough treatment, while the root bark of *Ardisia crenata* Sims and *Ardisia crispa* (Thunb.) A. DC. were employed for treat sore throat treatment. Apricot leaves and Saposhnikovia divaricata were used to treat diarrhea, with local residents believing in their high efficacy.

There are eight types of traditional medicinal formulations made from the 187 medicinal plants used by the Gelao people. Among them, decoctions are the most commonly used form, with 120 different types of decoctions that can be prepared by boiling in water or brewing as tea. Other formulations include ointments (21), tinctures (19), powders (8), infusions (7), fumigants (2), pills (2), plasters (1), and 19 others that could not be classified (Figure 7B).

### Functions and applications of traditional medicinal plants used by the gelao ethnic group

**FIGURE 8:**
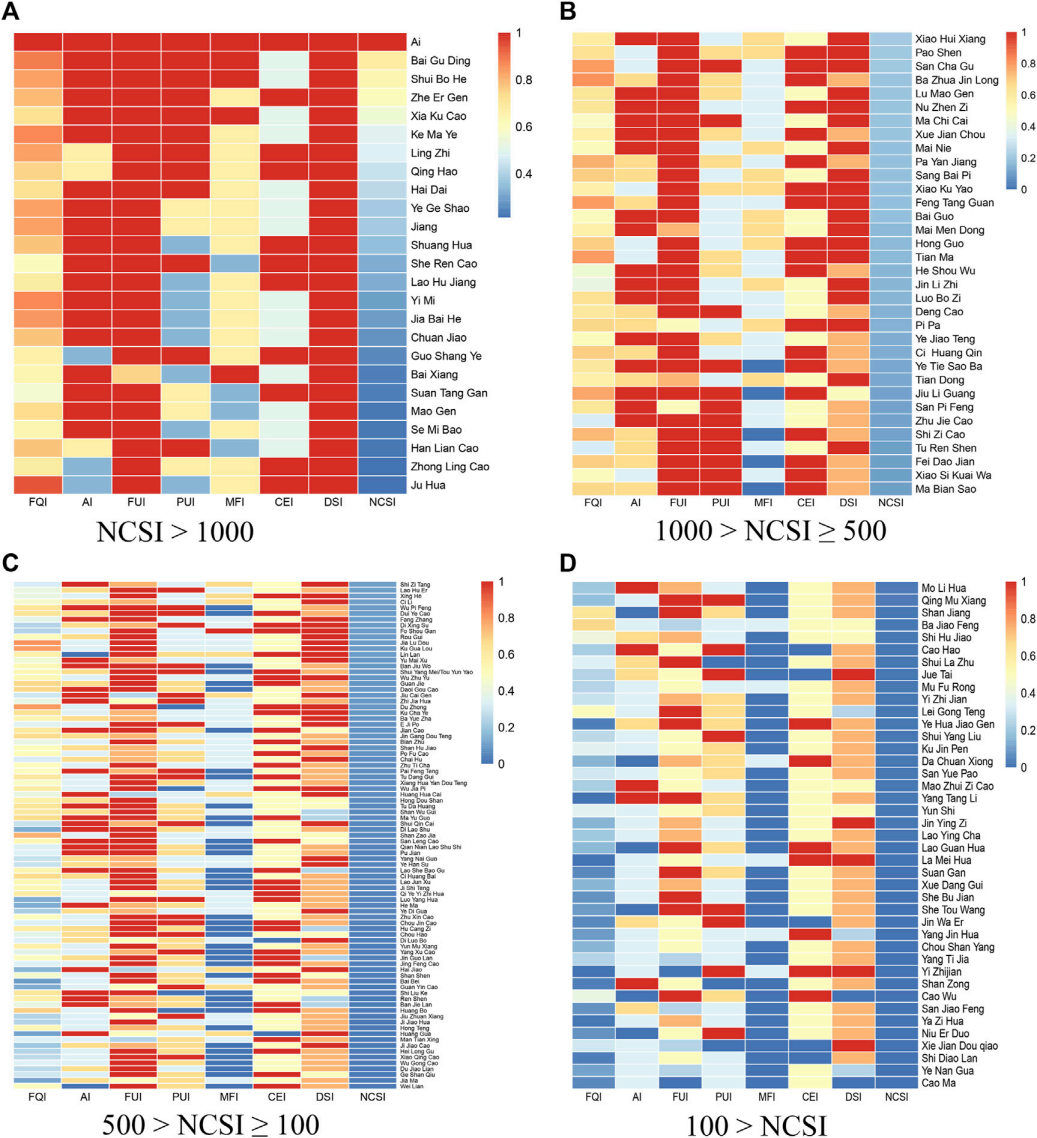
Quantitative evaluation of traditional Gelao medicinal plants. **(A)** NCSI >1000. **(B)** 1000 > NCSI ≥500. **(C)** 500 > NCSI ≥100. **(D)** 100 > NCSI.

We employed quantitative ethnobotanical methods to assess the significance of 187 traditional medicinal plants used by the Gelao ethnic group. The results, comparing the importance index (NCSI) of Gelao traditional medicinal plants, are presented in Supplementary Table S2. Based on the NCSI, we categorized the Gelao traditional medicinal plants into clusters and identified those that are widely utilized, highly valued, and play significant roles in Gelao traditional healthcare. The first cluster (NCSI >1000) consists of 25 traditional medicinal plants, including *Artemisia argyi* H.Lév. & Vaniot, *T. mongolicum* Hand.-Mazz., *Mentha canadensis* L., *Houttuynia cordata* Thunb. and *Prunella laciniata* L., which are commonly encountered in daily life and are well-known among local residents. Moreover, many of them serve as both medicinal and edible resources. The second cluster (1000 > NCSI ≥500) comprises 34 traditional medicinal plants such as *Epimedium brevicornu* Maxim., *A. crenata* Sims, *Portulaca oleracea* L., and *T. paniculatum* (Jacq.) Gaertn. This cluster is important for daily healthcare among local residents and has unique usage methods with high development potential. The third cluster (500 > NCSI ≥100) includes 88 traditional medicinal plants, which exhibit a diverse range of species and are mainly employed to treat common and special diseases in the local area. Although this cluster has relatively lower levels of development, it holds promising prospects for further exploration. The fourth cluster (100 > NCSI) encompasses 40 traditional medicinal plants, which obtain lower importance indices not due to their therapeutic effects or application value, but because they are mainly utilized for treating rare diseases among the Gelao population or possess certain toxic side effects (Figure 8).

## Discussion

In rural areas of China, traditional herbal doctors serve as the first line of defense for residents’ health, and even in modern times with highly developed medicine, they still play a vital role (Wei et al., 2017). While influenced by traditional Chinese medicine, most ethnic herbal doctors have incorporated their own theories and experiences, gradually blending their unique characteristics with the broader medical practices. This integration has not only preserved their traditional knowledge but also improved their medical skills.

### Application characteristics of traditional gelao herbal medicine

The Gelao ethnic group resides in a mountainous region with complex geological structures (Liu, 2007; Zheng, 2007). The area experiences a subtropical humid climate with warm and moist conditions, along with abundant rainfall. However, scarcity of agricultural resources, outdated traditional farming practices, and various environment, climate, geology, resources, and cultural factors have contributed to the development of distinct applications for preventing and treating common diseases among the Gelao people. Due to the high acidity of the soil in the region, as well as the tendency towards oily, greasy, raw and cold foods, and the humid and rainy climate, gastrointestinal disorders, rheumatism, and traumatic injuries are the most common diseases among the Gelao people.

The Gelao people often use *Pimpinella candolleana* Wight et Arn. To treat various gastrointestinal diseases. They mash the fresh *Pimpinella candolleana* Wight et Arn together with rice water and consume the juice. We have conducted pharmacological experiments to validate its significant therapeutic effects on gastrointestinal disorders. Although the Chinese Pharmacopoeia stipulates that the medicinal part of *A. crenata* Sims is dry root, the Gelao people use fresh or dried root bark to treat various inflammations (such as gingivitis and tonsillitis). To investigate this characteristic usage, we tested the content of the main effective active ingredient, bergenin, in the root and bark of *A. crenata* Sims, and found that it mainly exists in the bark, with a very low content in the wood part (these results will be reported in other articles).

Due to the damp climate and living conditions, the Gelao people have a relatively high incidence of rheumatic diseases, especially among the elderly. To alleviate rheumatic problems, they frequently use spicy condiments like *Capsicum annuum* L. and *Zanthoxylum bungeanum* Maxim., known for their moisture-removing properties. In terms of medicinal treatment, they use various vine-based herbs and animal-based medicines, either externally as poultices or by preparing wines with snake medicines. Regarding respiratory diseases, due to the high humidity and low temperature in the region during winter, people generally use coal or wood for heating, leading to poor indoor air circulation and making them susceptible to respiratory ailments such as colds, coughs, and asthma. To address these issues, the Gelao people utilize specific herbs like *S. japonica* (Blume) Miq., *R. carnea* (Andrews) Kunth, *Ophiopogon japonicus* (Thunb.) Ker Gawl., and *Adenophora stricta* Miq. To make tea or mix with egg whites to alleviate symptoms. In the case of skin diseases, as people generally live in mountainous areas where mosquito bites are common, they rely on various herbs such as *Senecio scandens* Buch.-Ham. ex D. Don, *Verbena officinalis* L., and *A. argyi* H.Lév. & Vaniot to create herbal baths for treating skin diseases such as eczema, scabies, and acne.

The Gelao people highly value fresh herbal medicine. Traditional Gelao herbal doctors typically collect herbs themselves from the mountains, with only a few herbs purchased from the market. These herbal doctors also cultivate commonly used herbs around their clinics, often within their own courtyards, resulting in up to thirty different species being present. This practice is not solely for convenience but primarily driven by the Gelao herbal doctors’ strong sense of preserving precious herbal resources. In fact, the traditional Gelao herbal medicine also includes abundant animal and mineral medicines, such as cinnabar in mineral medicine, and *Passer montanus*, Pheretima, Elephant Leather in animal medicine (Wang, 2017). However, due to various factors, the Gelao people mainly use herbal medicines. Overall, the distinctive applications of these herbal medicines reflect the Gelao people’s wisdom and their long-standing tradition of utilizing traditional herbal medicine.

### Comparison of traditional medicinal plants of the gelao ethnic group with other ethnic groups

Firstly, the sources of plants are different. Traditional healers often use readily available medicinal plants in their surroundings to treat diseases (Hong et al., 2015b). This phenomenon is influenced by the specific environment in which these groups reside (Liu et al., 2016). For example, the Tibetan people inhabit high-altitude areas, resulting in the utilization of medicinal plants with characteristics specific to plateau regions (Li et al., 2016). Previous research conducted in the multi-ethnic regions of Gansu, Inner Mongolia, and Ningxia in northwest China showed that the significant drought and cold resistance of the traditional medicinal plants utilized in those areas (Jia et al., 2022). In contrast, The Gelao people predominantly inhabit the northern part of Guizhou Province, southwestern China, characterized by abundant rainfall, a mild climate, and diverse topography. This unique geographical environment provides a favorable living environment for plants and serves as a rich source of medicinal resources for Gelao traditional healers (Zeng et al., 2013). However, these medicinal plants obviously have a “southern China” characteristic, and traditional Chinese medicines such as ephedra, astragalus, and dried hay are rarely used by Gelao healers. Furthermore, there is limited overlap between the medicinal plants used by Gelao people and those employed by other ethnic groups, such as the Tibetan, Hui, Mongolian, and Uyghur people residing in the arid regions of northern China (Guo et al., 2022). For example, our survey of traditional medicinal plants in the multi-ethnic areas at the junction of Gansu, Ningxia, and Inner Mongolia revealed minimal similarities with those employed by the Gelao people.

Secondly, the way of inheritance is different. Compared with ethnic groups such as the Han, Tibetan, Mongolian, Zhuang, and Uyghur people, who have established complete medical education systems (Liang et al., 2016), Gelao healers are mainly engaged in agricultural production, with medical treatment as a secondary profession. This “agricultural-medical integration” mobile diagnosis and treatment population greatly limits the inheritance of Gelao medicine. Gelao traditional medicine is mainly passed down through family and apprenticeship, through methods such as “oral transmission and heart-to-heart teaching,” “Hands-on guidance,” and “exchange of medicinal formulas”. However, these practices have hindered the development and formalization of Gelao traditional medicine.

Thirdly, the theoretical system is different. Traditional medicine among ethnic groups such as the Han, Tibetan, Mongolian, Zhuang, and Uyghur people has formed a systematic theoretical system. In contrast, Gelao healers are still in the stage of empirical use of medicine. Therefore, the medicinal plants they use are often based on specific diseases, while they lack understanding of disease etiology and pathogenesis. Although they also emphasize treating the root cause of the disease, they primarily focus on treating specific symptoms, and their treatment of mental illness is closer to witchcraft. On the other hand, Gelao people also emphasize balancing the body’s energy. Some Gelao healers believe that most diseases can be treated by regulating the body’s energy, and they believe that the medicinal plants they use can provide useful energy to patients. When the body’s energy is balanced, the disease will naturally disappear. Similarly, traditional medicine in other regions or ethnic groups in China also emphasizes the balance of the body’s “yin” and “yang”, and the five elements to achieve therapeutic outcomes (Wang et al., 2022).

### Challenges and threats to the use of traditional medicinal plants among the gelao ethnic group

Traditional medicinal plants used by the Gelao ethnic group have demonstrated significant therapeutic effects in certain unique diseases within their local region. However, they also face numerous challenges, including imprecise dosages, unregulated use, and hygiene and safety issues. Our research revealed that traditional Gelao herbalists lack accurate measuring tools when preparing herbal remedies, often relying on subjective estimation. Hygiene issues arise from the rudimentary conditions of their “pharmacies”, which frequently result in mold growth on the medicinal plants. Furthermore, the processing of toxic herbs is often insufficiently regulated, posing significant risks to patient safety. Although Gelao herbalists possess extensive knowledge of the properties and side effects of toxic herbs, they often use these remedies externally or prescribe them for long-term consumption with explicit instructions for preparation. Additionally, most Gelao herbalists have unique formulas and primarily treat two to five specific diseases.

Based on the challenges and limitations of the Gelao traditional medicinal plants. We believe that the Gelao traditional medicinal plant resources are threatened by ecological environmental changes, human destruction, alien invasion, and other factors, resulting in some plant species becoming rare or extinct. The traditional medicinal plant knowledge is affected by cultural inheritance gaps, education deficiencies, market competition, and other factors, resulting in some knowledge being forgotten or lost. The scientific value and social value of the Gelao traditional medicinal plants have not been fully recognized and utilized, resulting in some plants and knowledge being inefficiently or excessively used. The local government has also made some efforts based on this. The government has strengthened the protection and management of the Gelao traditional medicinal plant resources, established some facilities such as nature reserves, botanical gardens, seed banks, etc., implemented some policies such as returning farmland to forest, ecological compensation, green development, etc. It has strengthened the inheritance and popularization of the Gelao traditional medicinal plant knowledge, carried out some activities such as ethnic education, cultural heritage, science popularization, etc., and cultivated some talents such as ethnic pharmacists, ethnic scholars, ethnographers, etc. It has strengthened the research and development of the Gelao traditional medicinal plants, supported some work such as scientific research projects, technology transfer, product innovation, etc., and promoted some industries such as ethnic medicines, ethnic foods, ethnic tourism, etc.

### The current status of traditional medicinal knowledge among the gelao ethnic group and the importance of preserving traditional herbal knowledge

Our research on traditional consumption of wild plants by the Gelao ethnic group revealed that their traditional cultural knowledge is gradually being eroded by Han culture and globalized digital culture (Guo, 2018). Traditional medicinal knowledge among the Gelao ethnic group is particularly vulnerable in this regard (Xie et al., 2022). The valuable information provided by our interviewees mostly came from elderly and middle-aged individuals, with the latter representing the newest generation of Gelao herbalists, most of whom are over 45 years old. Younger Gelao herbalists have become almost extinct, and the few remaining young practitioners have typically received systematic training in traditional Chinese medicine and lack the distinct characteristics of traditional Gelao herbalists. They mostly rely on purchased Chinese medicine pills rather than gathering their own herbs. While they have abandoned some of their ancestors’ negative practices, they have also lost some of their ethnic characteristics. Additionally, male interviewees were more likely to provide valuable information than females, who provided fewer details about the types of herbs, processing methods, and usage techniques. This may be related to the traditional Gelao custom of passing herbal knowledge down to males but not to females.

The unique characteristics of traditional Gelao medicine are being gradually absorbed by Chinese and modern medicine cultures, with much of the mystical knowledge of traditional Gelao medicine now relegated to folklore. This cultural erosion is accelerating, and many traditional cultural practices now only exist in museums and ancient texts. Without proper documentation, the accumulated knowledge of generations of traditional medicine practitioners will be lost forever (Mohammed et al., 2019; Xiong et al., 2020; Shu et al., 2022).

### Limitation and prospect of this study

This study also has some limitations. It only involves the ethnobotanical aspect of the Gelao traditional medicinal plants, without involving the ethnopharmacological aspect, that is, it did not conduct chemical and pharmacological analysis of these plants to verify their active ingredients and mechanisms of action. The study only relies on the subjective reports of the Gelao people on plant use and knowledge, and the number of informants is also small. Since this study only focuses on the use of medicinal herbs by the Gelao ethnic group, it is biased towards the field of ethnobotany. We will also conduct more in-depth chemical and pharmacological studies in our future research, in order to provide valuable clues for further development and utilization of new and valuable Gelao medicines, as well as more scientific support for Gelao traditional medicine.

## Conclusion

The Gelao community have accumulated wealth of experience in utilizing herbal medicine to combat diseases. We have collected important information on 187 medicinal plants, including their botanical names, sources, processing methods, primary therapeutic uses, and administration techniques. However, we have also observed a rapid decline in this valuable knowledge. Our mission is to document, systematize, and safeguard these clinically validated medicinal practices. This undertaking opens up avenues for the preservation of traditional herbal medicine knowledge among the Gelao people while offering firsthand insights for the development and utilization of herbal remedies with notable therapeutic effects against specific ailments. Nevertheless, it is important to acknowledge the potential bias in the information we have obtained, as well as the presence of unverifiable or inaccurate data from our sources, which may introduce certain inaccuracies into our analysis.

Furthermore, the information we have gathered is derived from the medication experience of local residents, and further research is necessary to verify the reliability, safety, and efficacy of these medicines in clinical applications. In the future, we plan to conduct comprehensive and systematic investigations into Gelao traditional medicine and culture, establish appropriate protective measures, and develop high-value pharmaceutical products.

## Data Availability

The original contributions presented in the study are included in the article/Supplementary Material, further inquiries can be directed to the corresponding authors.

## Abbreviations

PRA, participatory rural appraisal; 5W + 1H, Why What Where When Who How; FQI, frequency of quotation index; AI, availability index; FUI, frequency of utilization index; PUI, parts used index; MFI, multifunctional use index; CEI, curative effect index; DSI, drug safety index; NCSI, national plant cultural significance index.
